# Molecular Dynamics Simulation on Thin-Film Lubrication of a Mixture of Three Alkanes

**DOI:** 10.3390/ma13173689

**Published:** 2020-08-20

**Authors:** Run Du, Anying Zhang, Zhihua Du, Xiaoyu Zhang

**Affiliations:** 1 School of Mechanical Engineering, Southwest Jiaotong University, Chengdu 610031, China; azhang@mechplus.com (A.Z.); zdu@mechplus.com (Z.D.); zhangyu3035@126.com (X.Z.); 2 Technology and Equipment of Rail Transit Operation and Maintenance Key Laboratory of Sichuan Province, Chengdu 610031, China

**Keywords:** thin film lubrication, molecular dynamics, density stratification, mixture, alkane

## Abstract

We used the COMPASS forcefield to perform molecular dynamics (MD) simulation of a mixture composed of three alkanes as the lubricant for the thin-film lubrication. The viscosity of the lubrication film in the non-working state, the final film thickness, and density distribution were investigated. The results reveal that the viscosity error among different initial film thicknesses in the non-working state is within 5%, which confirms the applicability of the model and the forcefield. The viscosity decreases oscillating as temperature increases. Whatever the initial film thickness is, the film thickness change rate with respect to pressure load is almost the same. When pressure increases, the density peaks increase. As the initial film thickness increases, the normalized thicknesses of adsorption and ordered layers decrease. In nanoscale, the density predicted by the MD simulation is higher than the prediction of the Tait equation, even if the adsorption layers is excluded.

## 1. Introduction

As lubrication dimension tends to nanoscale, many fascinating phenomena are revealed, which are much different from the Elasto-Hydrodynamic Lubrication (EHL) and the Hydrodynamic Lubrication (HDL). The special arrangement of nanoscale molecules, the friction, film thickness, and viscosity of the lubricant on the nanoscale are different from those of EHL and HDL [1,2]. If the film thickness is less than 15 nanometers, the relationship between the film thickness and the speed is abnormal [3]. In the state of thin film lubrication, the lubrication film delaminates [4,5,6].

Molecular dynamics (MD) simulation is a popular method for studying nano-lubrication. The significant properties of such molecular systems can be predicted accurately by MD simulation with both ab initio and empirically parameterized classical forcefields under ambient conditions, and the results can be presented intuitively [7].

Through MD simulation, slip phenomenon and uneven density distribution have been widely recognized. The wall speed, the strength of the avoiding surface adsorption, and the wall roughness have important effects on the speed slip [8]. Savio et al. [9] found that the wall slip phenomenon is related to the length of the lubricant molecular chain in addition to the strength of the wall adsorption, and the slip speed of the long-chain alkane on the wall is lower than that of the short-chain alkane. Gkagkas and Ponnuchamy [10] studied the wall slip of ionic and non-ionic liquids, and the results show that both have obvious slips, but the slip velocity curve of ionic liquids is flatter than that of non-ionic liquids. For the density stratification phenomenon, Luo et al. [5,6] first proposed the thin film lubrication stratification theory through experiments, which was then confirmed by many researchers via MD simulations. Pan and Gao [11,12] studied the density distribution and compressibility of PEC7 molecules under different temperature, pressure, and initial film thickness conditions. It is pointed out that the density distribution is oscillating. However, these studies only compared the results of three different initial film thicknesses, and the film thickness span is less than 10 nanometers. By studying the density distribution of alkanes with different chain lengths, Zheng et al. [13] pointed out that increasing the chain length helps to form a lubricating layer of a single molecular layer and makes the density peak increase. Thus, lubricating materials play a vital role in the characteristics of lubrication [14,15,16,17,18]. This research indicates that there are many factors that affect the lubrication characteristics of the film. It is necessary to explore these influencing factors deeply and extensively in order to understand the thin film lubrication more comprehensively.

In addition, the forcefield is the key foundation for MD simulation. Ewen et al. [7] studied the accuracy of different forcefields on the basis of density and viscosity. They pointed out that the all-atom forcefield predicts the viscosity more precisely than the united-atom forcefield. Thus, they recommended the all-atom forcefield. Pan et al. [19] studied the layered structure of three naphthenes using the united-atom forcefield and found that the layering is obvious. Galvani Cunha and Robbins [20] conducted a non-equilibrium MD simulation of the viscosity of 2,2,4-trimethylhexane at different pressures using an all-atom forcefield. The study reveals that even if under high pressure, the viscosity exceeds the limit of Newtonian viscosity, and the deviation between MD simulation and experimental results is less than 40%. Martini et al. [21] also found that the viscosity is shear-thinning as the film thickness decreases while the united-atom forcefield is used. It is clear that forcefields significantly influence the density distribution, viscosity, and film structures.

However, most liquid lubricants are mixtures. Most researchers focus on single-component alkanes, while some studied two-component lubricants [22,23]. Few have carried out research about mixtures that are composed of more than two components. In order to close the gap, we built an MD model of a mixture composed of three alkanes and studied its properties, including viscosity in the non-working state, final film thickness, and density distribution, while the film thickness is in the range of a few nanometers to more than ten nanometers.

## 2. The Model and Methodology

### 2.1. Model

**Figure 1:**
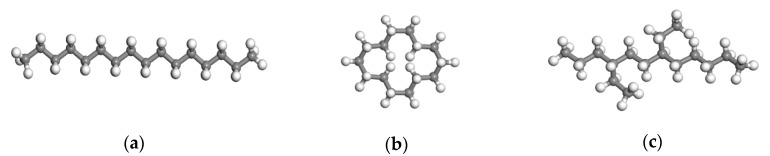
The all-atomic model of three molecules in the lubricant: (**a**) N-hexadecane molecule; (**b**) cyclotetradecane; (**c**) 4,7-diethyldodecane.

**Figure 2:**
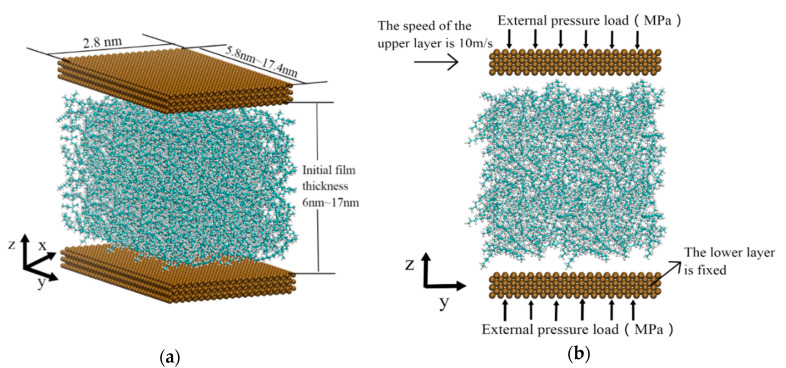
The lubrication system model: (**a**) the perspective view; (**b**) the load of the lubrication system.

Liquid lubricants generally are composed of a base oil and additives. The base oil is the main component of the lubricant, which determines the basic properties of the lubricating oil. The alkanes are the main components of the base oil, including n-alkanes, branched alkanes, and naphthenes. In this paper, the lubricant was a mixture, which was composed of three alkane molecules: N-hexadecane (C_16_H_34_) straight chain molecules, cyclotetradecane (C_14_H_28_), and 4,7-diethyldodecane (C_16_H_34_) with 12 carbon atoms on the backbone and two ethyl side branches. Their all-atomic models are shown in Figure 1. In the simulation system shown in Figure 2a, their number of molecules was the same, and the mass percentages of N-hexadecane, cyclotetradecane, and 4,7-diethyldodecane were 34.9%, 34.9%, and 30.2%, respectively.

As shown in the Figure 2a, the lubrication system was divided into three layers. Both upper and lower layers were Fe atoms corresponding to the metal parts. Each consisted of three strata of Fe atoms in the body-centered cubic (BCC) () lattice (the lattice constant of Fe is 2.867 Å). The middle layer was the lubricant obtained by random filling of alkane molecules. The length along x-axis was fixed as 28 Å. The sizes along the y-axis were 58 Å, 87 Å, 116 Å, 145 Å, and 174 Å. The initial film thicknesses (*h*_0_) along the z-axis were 60 Å, 80 Å, 110 Å, 140 Å, and 170 Å. The thickness of Fe layers was 9 Å.

The boundaries are shown in the Figure 2b. Along x and y directions, periodic boundary conditions were applied to ensure a constant number of molecules in the system during the simulation. The outer surfaces of upper and lower layers were subjected to the external pressure load. The lower layer was fixed. The atoms of the upper layer moved collectively. The velocity was 10 m/s along the positive direction of y-axis.

The initial density was *ρ*^0^ = 0.8 g/cm^3^. The system temperature was set to *T* = 300 K in most of the simulations, or it is explicitly stated.

### 2.2. Forcefield

Considering the consistency of simulation and practical applications, each alkane molecule was simulated using the all-atom Condensed-phase Optimized Molecular Potentials for Atomistic Simulation Studies (COMPASS) forcefield [24], which can effectively simulate molecular systems, such as organic polymers and inorganic molecules. COMPASS includes potential energy models with the bonded potentials (*E_B_*, *E_A_*, *E_D_*, *E_I_*) and the non-bonded potentials (*E_LJ_*). The functional forms adopted in COMPASS are the same as those used in Consistent Force Fields (CFFs). However, the difference between CFFs and COMPASS is the parameters for alkanes in the LJ-9-6 (non-bond potential) function. In LJ-9-6 function, for solving the interactions of the atoms belonging to different molecules and also those of the atoms of the same molecule, the cutoff distance *r_c_* is 1.0 nm (about 2.5 times $r_{ij}^{o}$, the LJ length scale of the atom–atom interaction). For interactions of atom pairs, the 6th order combination law [25] was used to calculate the off-diagonal parameters:

(1) $$E_{\mathrm{LJ}}=\varepsilon_{ij}\left[2{\left(\frac{r_{ij}^{o}}{r_{ij}}\right)}^{9}-3{\left(\frac{r_{ij}^{o}}{r_{ij}}\right)}^{6}\right],$$

(2) $$r_{ij}^{o}={\left(\frac{{\left(r_{i}^{o}\right)}^{6}+{\left(r_{j}^{o}\right)}^{6}}{2}\right)}^{1/6},$$

(3) $$\varepsilon_{ij}=2\sqrt{\varepsilon_{i}\cdot \varepsilon_{j}}\left(\frac{{\left(r_{i}^{o}\right)}^{3}\cdot {\left(r_{j}^{o}\right)}^{3}}{{\left(r_{i}^{o}\right)}^{6}+{\left(r_{j}^{o}\right)}^{6}}\right).$$

For the interaction of atoms, the parameters are given in Table 1.

Table 1 lists the types of atoms that produce paired effects. There were four types of atoms in the molecular structure of the lubricant. In N-hexadecane and cyclotetradecane, the carbon atom is *C*_3_, which is the generic *sp^3^* carbon. However, there are two types of carbon atoms in 4,7-diethyldodecane, *C*_3_, and *C*_43_, where *C*_43_ is the *sp^3^* carbon with three heavy atoms attached.

The metal layers used in this research were bcc lattice iron atoms. The Finnis-Sinclair embedded-atom method (FS-EAM) potential function [26] can more accurately predict the cohesive energy and the repulsive character of the bcc lattice, compared to LJ or Morse potentials. Therefore, the FS-EAM potential was employed.

### 2.3. Simulation Method

All simulations were performed using the Large-scale Atomic/Molecular Massively Parallel Simulator (LAMMPS, http://lammps.sandia.gov) [27]. The simulation process was divided into three stages. First, the simulation was run to make the system reach equilibrium before loading. Second, after the system relaxation simulation was completed, the system was loaded. The pressure load was achieved by applying equal forces to the upper and lower metal layers in opposite directions with the same magnitude. Pressure loads can be achieved by adjusting the applied equalizing force. Finally, the simulation was continued while the pressure loads kept constant, and the velocity was applied to the upper metal along the y-direction.

The system was divided into two groups: the iron atom group and the molecular group. The iron atom group was fixed during the relaxation stage. All groups followed the Nose-Hoover style non-Hamiltonian equations of motion. The equations were used to generate positions and velocities sampled from the canonical ensemble (NVT). The thermostatting was achieved by setting the same initial and end temperatures. The barostatting was achieved by setting the force on the wall surface, which were coupled to the particle velocities.

The lubricant viscosity in the non-working state was simulated with a pressure load of 0.1 MPa applied to the system. The viscosity was calculated via the Green-Kubo formula:

(4) $$\eta =\frac{V}{3K_{B}T}\int_{0}^{t}dt\sum_{\alpha }\sum_{\beta }\langle P_{\alpha \beta }\left(0\right)\cdot P_{\alpha \beta }\left(t\right)\rangle ,$$

(5) $$P_{\alpha \beta }=\frac{1}{V}\left[\sum_{i=1}^{N}m_{i}v_{i\alpha }v_{i\beta }+\sum_{i=1}^{N}\sum_{j>i}^{N}r_{ij\alpha }f_{ij\beta }\right],$$

where *η* is the shear dynamic viscosity, *V* is the system volume, *K_B_* is the Boltzmann constant, *T* is the system temperature, *P_αβ_* is the component of the pressure tensor *P* (*α*,*β* = *x*,*y*,*z*. but α is different from *β*), *m_i_* is the mass of the molecule *i*, v_ia_ is the velocity of molecule *i* in direction *α*, *r_ija_* is the distance between molecules *i* and *j* in direction *α*, and *f_ijβ_* is the intermolecular force between molecules *i* and *j* in direction *β*.

## 3. Results and Discussion

### 3.1. Viscosity

**Figure 3:**
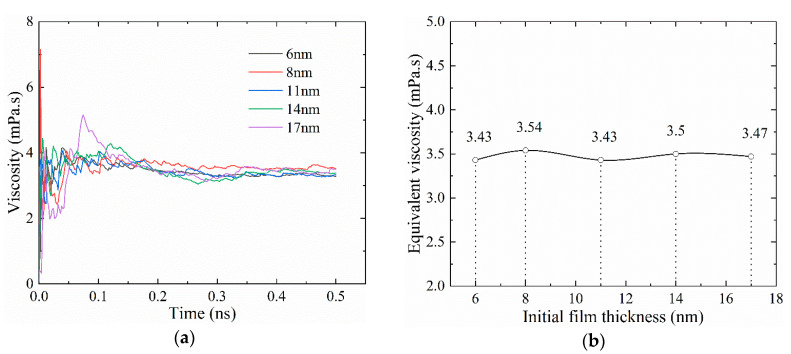
The lubrication film viscosity under the non-working state (*P* = 0.1 MPa, *T* = 300 K): (**a**) evolution of viscosity; (**b**) time-average viscosity of different initial film thickness.

Figure 3 gives the lubrication film viscosity in the non-working state. As Figure 3a shows, the viscosity of the lubrication film started to stabilize after 0.2 ns. The viscosity value ranged from 3 mPa·s to 4 mPa·s. The viscosity values of different initial film thicknesses are much closer. Figure 3b compares the time-average viscosity values of different initial film thicknesses after being stabilized. The average lubricant viscosity of different initial film thicknesses was 3.46 mPa·s. The viscosity difference between the ones of different initial film thicknesses, and the average one was within 5%, which shows that the viscosity is irrelevant to the initial film thickness in the non-working state.

**Figure 4:**
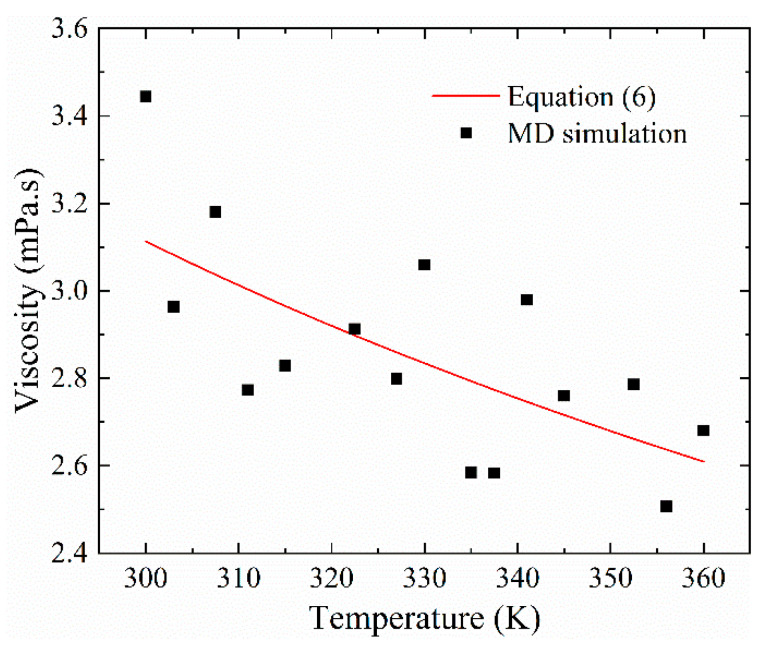
The lubrication viscosity vs. temperature (*P* = 0.1 MPa, *h*_0_ = 6 nm).

Figure 4 presents the lubrication film viscosity variation with temperature. As it shows, the viscosity of the lubrication film decreased oscillating as temperature increased. We have made a least-square fitting with the Walther equation; the viscosity varies with temperature as:

(6) $$\log(\log(\eta /\rho^{0}+0.7))=1.204-0.5583741\cdot \log(T),$$

where *η* is the viscosity in mPa·s, *ρ*^0^ is the density in g/cm^3^, and *T* is the temperature in K.

### 3.2. Film Thickness

**Figure 5:**
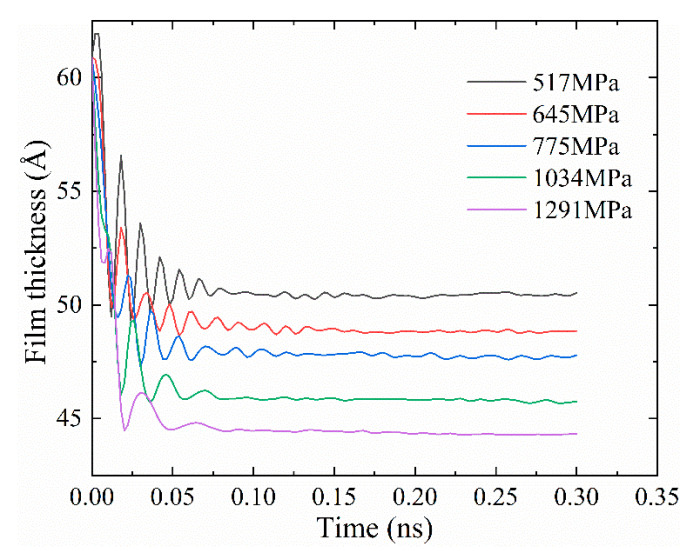
The lubrication film thickness under different pressure loads (*h*_0_ = 6 nm).

Figure 5 shows the thickness evolution of the lubrication film when *h*_0_ = 6 nm under different loads. It can be seen that the film thickness was stable after 0.1 ns. However, the film thickness curve after 0.1 ns was not completely smooth, because pressure and velocity loads were applied to it at the same time. This impulse loading causes vibration. As the simulation runs, the vibration is attenuated.

**Figure 6:**
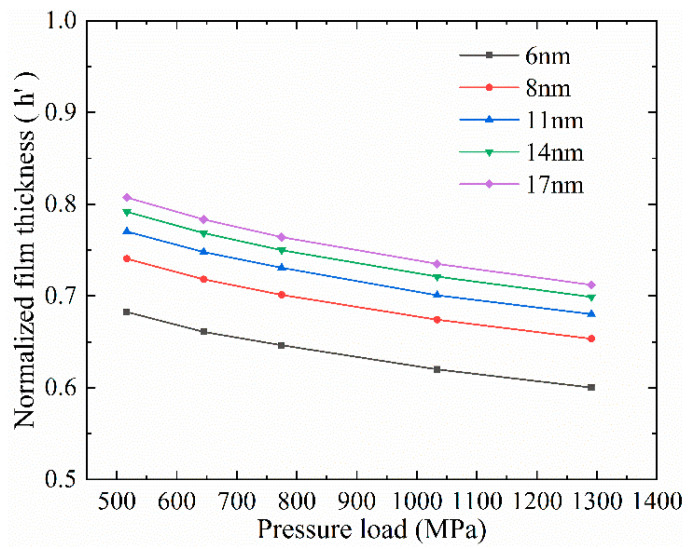
The dependence of film thickness on external pressure with different initial film thickness.

The change of lubrication film thickness under different pressure loads is presented in Figure 6. The normalized film thickness is *h’* = *h_l_*/*h*_0_, where *h_l_* is the film thickness under loads. It can be clearly seen that thickness of the lubrication film decreased almost linearly as the pressure increased. In addition, when the lubrication films were subjected to the same pressure load, the larger the initial thickness of the lubrication film, the smaller the film thickness reduction *h’*_d_ (*h’*_d_ = 1−*h’*). The negative correlation between the film thickness reduction *h’*_d_ and the initial film thickness *h*_0_ indicates that the film thickness is directly related to the bearing capacity of the lubrication film. The film with a larger initial film thickness tends to bear a larger load.

### 3.3. Density

**Figure 7:**
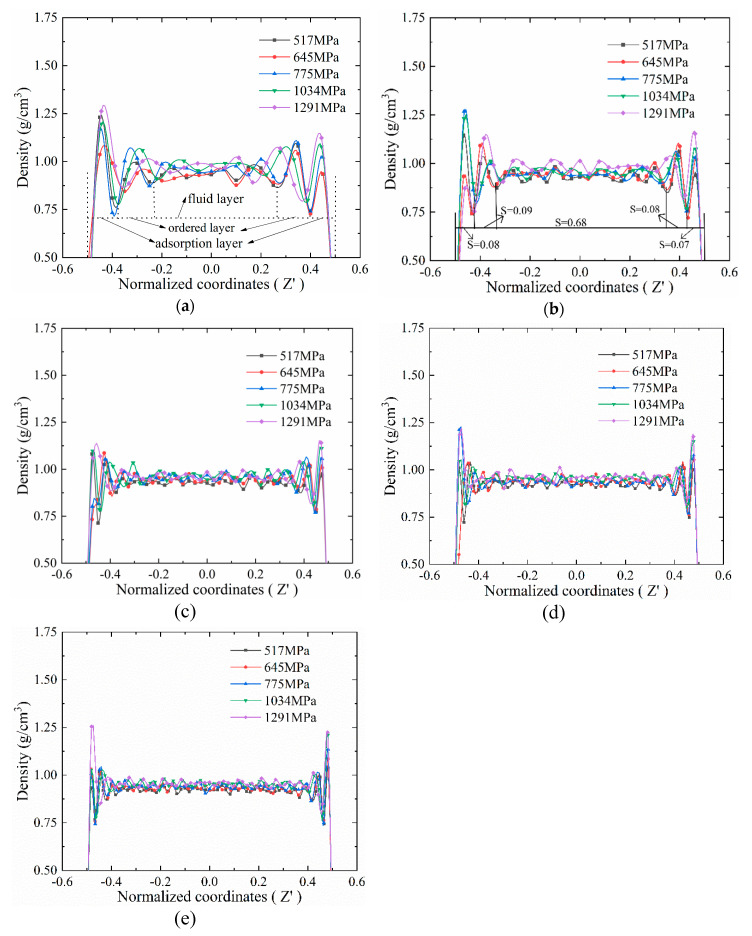
The density distribution curve of lubrication film with different initial film thickness: (**a**) *h*_0_ = 6 nm; (**b**) *h*_0_ = 8 nm; (**c**) *h*_0_ = 11 nm; (**d**) *h*_0_ = 14 nm; (**e**) *h*_0_ = 17 nm.

The density distribution of different initial film thicknesses under working conditions (*P* = 517, 645, 775, 1034, 1291 MPa and *v*_y_ = 10 m/s) is shown in Figure 7. In order to better understand the stratification, we also normalized the film thickness in the z direction; the normalized coordinates *Z*’ are as follows:

(7) $$Z^{\prime }=\frac{Z-\frac{Z_{\mathrm{up}}+Z_{\mathrm{low}}}{2}}{Z_{\mathrm{up}}-Z_{\mathrm{low}}},$$

where *Z*_up_ is the coordinate of the lubricating film close to the upper metal, and *Z*_low_ is the coordinate close to the lower metal. Hence, *Z*’ = 0 means the right middle of the film thickness.

As shown in Figure 7, it can be clearly seen that there was density stratification [6,28]. There were four peaks in the density curves, and the valley values divided the density curve into five segments. In the state of thin film lubrication, the lubrication film can be divided into three types of layers: the adsorption layer, the ordered layer, and the fluid layer [5,6], as shown in Figure 7a.

The adsorption layer is the outermost layer of the film on both sides. It forms because the adsorption force between the lubricant molecule and the metal atom is stronger than the adsorption force between the lubricant molecules. This causes the lubricant molecules to be adsorbed on the metal wall surface [5,6]. The thickness of the adsorption layer was the thinnest, but the density was the largest among these three layers. The normalized thickness S (*S* = *h*_a,o,f_/*h*_l_, *h*_a_, *h*_o_ and *h*_f_ represent the thickness of the adsorption layer, the ordered layer and the fluid layer, respectively) was used to measure the thickness of each lubricating layer. It can be seen from Figure 7a,b, when initial film thicknesses were 6 nm and 8 nm, the normalized thickness of the adsorption layer was *S* = 0.1. However, as shown in Figure 7c–e, the normalized thickness *S* < 0.1 when initial film thickness *h*_0_ = 11 nm, 14 nm, 17 nm, because the adsorption layer generally consists of 1–2 molecular layers. Therefore, when the initial film thickness increases, the normalized film thickness of the adsorption layer decreases. Under the action of shear force, the molecules in the adsorption layer tend to be distributed along the main chain direction perpendicular to the film thickness direction, and the molecules are arranged in parallel, tight and orderly. Thus, the density of the adsorption layer is much higher than ordered and fluid layers.

As shown in Figure 7a, the ordered layer is the peak next to the adsorption layer. The ordered layer is between the adsorption layer and the fluid layer. The degree of order of the ordered layer is affected by many factors, such as pressure, shear rate, and lubricant viscosity [5]. We inspected only the pressure here. As shown in Figure 7a,b, under different initial film thicknesses and external pressure load, the normalized film thickness of the ordered layer was *S* = 0.1–0.2. It was slightly larger than that of the adsorption layer. In addition, it was getting smaller as the initial film thickness increased. The structure of the ordered layer has the characteristics of both the adsorption film and the fluid film [5]. That is, from the side near the adsorption film to the side near the fluid film, the molecular arrangement structure is getting more and more cluttered and increasingly disordered, hence the density of the ordered layer is between the adsorption layer and the fluid layer.

As shown in Figure 7a, the fluid layer is in the middle of the film. The density of this layer was the smallest. Here, the attraction of metal atoms is much smaller than the attraction between molecules, and the molecular distribution is not dense enough. The molecular arrangement of the fluid layer is completely disordered. Furthermore, its normalized thickness was *S* = 0.5–0.8, which was the largest normalized film thickness. The increase of initial film thickness makes the normalized thickness of the fluid layer increase.

**Figure 8:**
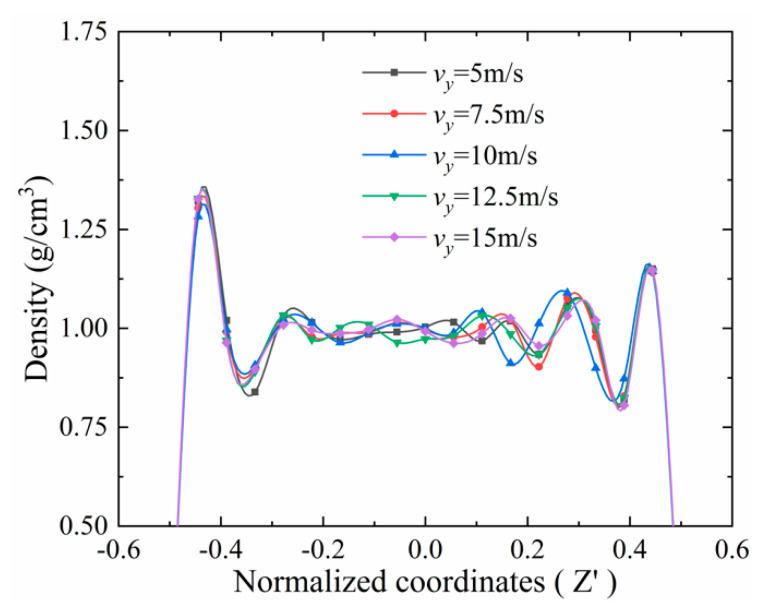
Density distribution of the film at different speeds (*h*_0_ = 6 nm, *P* = 1291MPa).

Figure 8 presents the density variation along z direction while the speed changes. As it shows, the density stratification was almost the same when the speed varies from 5 m/s to 15 m/s. This means the density stratification is irrelevant to the speed in the range of 5 to 15 m/s, when the initial film thickness is 6 nm. This is similar to the results obtained by Jabbarzadeh et al. [8], who found that when the shear rate is lower than 10^11.5^ s^−1^, the change in the shear rate does not cause a significant change in density. In this paper, the maximum shear rate of the case with *h_0_* = 6 nm was less than 10^10^ s^−1^. Furthermore, when the initial film thickness is larger than 6nm, the influence of speed is insignificant as well, because the shear rate decreases as the film thickness increases. Therefore, density stratification is much less sensitive to speed than pressure.

**Figure 9:**
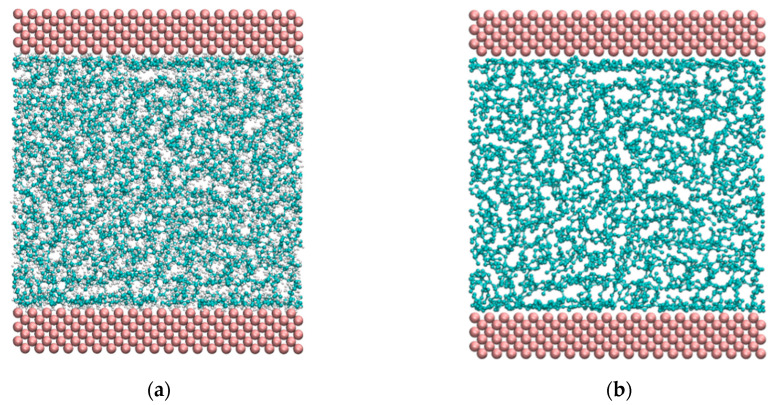
Snapshots at the end of the simulation (*h*_0_ = 6 nm, *P* = 1291 MPa): (**a**) snapshot of all atoms; (**b**) snapshot of iron atoms and carbon–carbon bonds remained.

In order to more intuitively observe the phenomenon of lubrication film delamination, molecular snapshots of initial film thickness *h*_0_ = 6 nm at the end of the simulation is shown in Figure 9. Figure 9a gives the snapshot of all atoms and Figure 9b snaps only both iron atoms and carbon–carbon bonds. The adsorption layer generally consisted of one molecular layer (Figure 9a,b). However, it is difficult to identify the ordered layer, although the density convex peak of the ordered layer can be found in the density distribution diagrams, as shown in Figure 7. The reason for difficult identification is that the density curve is the average of the statistical time and space in the simulation process. However, the snapshot only captures a certain moment, thus it is difficult to ensure that the snapshot matches the statistical results.

When the number of lubricant molecules is the same, the increase of the external pressure definitely causes the density to increase. As shown in Figure 7, the greatest density peaks of different initial film thicknesses generally increased as external pressure load increased. In order to reveal the relationship between the density of the lubrication film and the pressure, we compared the density value of the MD simulation with the Tait equation. The Tait equation is generally considered to be the best correlation for the high-pressure liquid phase density of a fluid [22]. The equation is as follows:

(8) $$\rho \left(T,P\right)=\frac{\rho^{o}\left(T\right)}{\left\{1+C\left(T\right)\ln\left[\left(B\left(T\right)+0.1\right)/\left(B\left(T\right)+P\right)\right]\right\}},$$

(9) $$\rho^{o}\left(T\right)=\rho_{0}^{o}+\rho_{1}^{o}T,$$

(10) $$C\left(T\right)=C_{0}+C_{1}T,$$

(11) $$B\left(T\right)=B_{0}+B_{1}T.$$

The Tait equation contains six adjustable parameters ($\rho_{0}^{o}$, $\rho_{1}^{o}$, *C*_0_, *C*_1_, *B*_0_, *B*_1_), which can be adjusted according to different fluid materials. Although the lubricant studied in this paper was a mixture, its compositions were similar to the single component N-hexadecane. Therefore, N-hexadecane was selected as a reference. The parameters of Tait equation are listed in Table 2.

**Figure 10:**
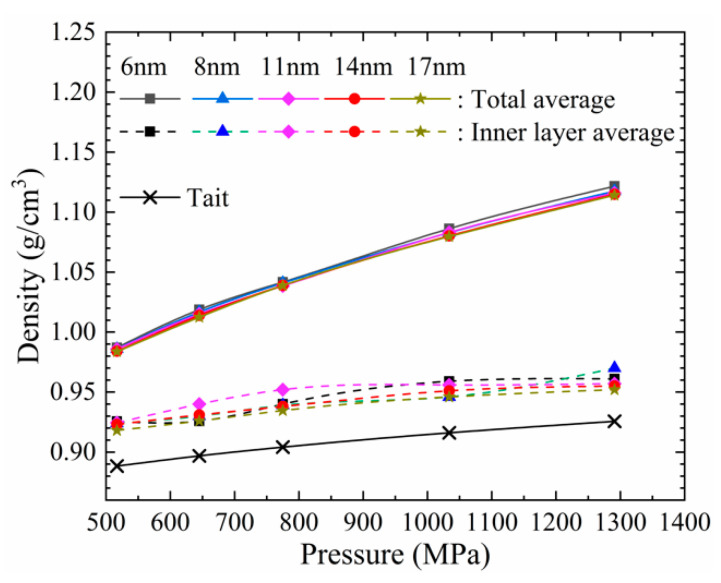
Density predictions of MD simulation and the Tait equation.

Figure 10 gives the density variation with pressure. As seen in Figure 10, as the pressure increased, the density value of the MD simulation increased. However, the MD result was much larger than the result of the Tait equation. However, the average density of inner layers (the ordered and the fluid layers, dash lines in Figure 10) was higher than the prediction of the Tait equation and lower than the total average density. Which means that the Tait equation cannot precisely predict the density in the nano lubrication state while the pressure is from 517 to 1291 MPa, even if in the ordered and fluid layers.

As the initial film thickness decreases, the molecular layers decrease, and the ordered and fluid layers become a smaller portion of the total molecular layers. Both of the layers were compressed when it is subjected to large pressure. However, as shown in Figure 7, the adsorption layer contributed much more to the fluid compressibility. The adsorption layer is much more influenced by pressure than ordered and fluid layers.

## 4. Conclusions

In this article, we focused on the dependence of the density distribution of the mixed alkane lubricant film on the initial film thickness and pressure load using MD simulation, while an all-atomic forcefield was used. Analyses of the non-working viscosity and the density delamination of the mixed alkane are carried out.

The results show that the viscosity of the mixed lubricant does not depend on the initial film thickness in the non-working state. This confirms the applicability of the COMPASS forcefield and the feasibility of the lubrication model. However, the viscosity decreases oscillating as temperature increases.

There is a stratification phenomenon while the initial film thickness ranges from 6 to 17 nm, but the density and normalized thickness of different lubricating layers are related to the initial film thickness and pressure. In general, when pressure increases, the density delamination is intensified: the density peaks increase. As the initial film thickness increases, the density delamination is weakened: the normalized thicknesses of adsorption and ordered layers decrease.

In nanoscale, the density predicted by the Tait equation is smaller than the prediction of MD simulation, even if the adsorption layers are excluded.

## Figures and Tables

**Table 1:** Parameters of LJ potential in COMPASS [24].

Atomic Category	*r^o^* (Å)	*ε*(kcal/mol)
Fe	2.6595	13.88920
C_43_	3.854	0.0400
C_3_	3.854	0.0620
H	2.878	0.0230

**Table 2:** N-hexadecane parameter of Tait equation [22].

Parameter	Unit	Value
$\rho_{0}^{o}$	kg m^−3^	9.7188 × 10^2^
$\rho_{1}^{o}$	kg m^−3^ K^−1^	−6.8027 × 10^−1^
*C*_0_	−	−1.2327 × 10^−1^
*C*_1_	K^−1^	5.5227 × 10^−^^4^
*B*_0_	MPa^−1^	−1.3895 × 10
*B*_1_	MPa^−1^ K^−1^	1.8296 × 10^−1^
